# New N4-Donor Ligands as Supramolecular Guests for DNA and RNA: Synthesis, Structural Characterization, In Silico, Spectrophotometric and Antimicrobial Studies

**DOI:** 10.3390/molecules28010400

**Published:** 2023-01-03

**Authors:** Ernest Ewert, Izabela Pospieszna-Markiewicz, Martyna Szymańska, Adrianna Kurkiewicz, Agnieszka Belter, Maciej Kubicki, Violetta Patroniak, Marta A. Fik-Jaskółka, Giovanni N. Roviello

**Affiliations:** 1 Faculty of Chemistry, Adam Mickiewicz University in Poznań, Uniwersytetu Poznańskiego 8, 61-614 Poznań, Poland; 2 Institute of Bioorganic Chemistry, Polish Academy of Science, Noskowskiego 12/14, 61-704 Poznań, Poland; 3 Institute of Biostructures and Bioimaging, Italian National Council for Research (IBB-CNR), Area di Ricerca Site and Headquarters, Via Pietro Castellino 111, 80131 Naples, Italy

**Keywords:** Schiff base, coordination polymers, Cu(I), Ag(I), molecular docking, CT-DNA, RNA

## Abstract

The present work reports the synthesis of new N4-donor compounds carrying p-xylyl spacers in their structure. Different Schiff base aliphatic N-donors were obtained synthetically and subsequently evaluated for their ability to interact with two models of nucleic acids: calf-thymus DNA (CT-DNA) and the RNA from yeast Saccharomyces cerevisiae (herein simply indicated as RNA). In more detail, by condensing p-xylylenediamine and a series of aldehydes, we obtained the following Schiff base ligands: 2-thiazolecarboxaldehyde (**L1**), pyridine-2-carboxaldehyde (**L2**), 5-methylisoxazole-3-carboxaldehyde (**L3**), 1-methyl-2-imidazolecarboxaldehyde (**L4**), and quinoline-2-carboxaldehyde (**L5**). The structural characterisation of the ligands **L1**-**L5** (X-ray, ^1^H NMR, ^13^C NMR, elemental analysis) and of the coordination polymers **{[CuL1]PF_6_}_n_** (herein referred to as **Polymer1**) and **{[AgL1]BF_4_}_n,_** (herein referred to as **Polymer2**, X-ray, ^1^H NMR, ESI-MS) is herein described in detail. The single crystal X-ray structures of complexes **Polymer1** and **Polymer2** were also investigated, leading to the description of one-dimensional coordination polymers. The spectroscopic and in silico evaluation of the most promising compounds as DNA and RNA binders, as well as the study of the influence of the 1D supramolecular polymers **Polymer1** and **Polymer2** on the proliferation of *Escherichia coli* bacteria, were performed in view of their nucleic acid-modulating and antimicrobial applications. Spectroscopic measurements (UV–Vis) combined with molecular docking calculations suggest that the thiazolecarboxaldehyde derivative **L1** is able to bind CT-DNA with a mechanism different from intercalation involving the thiazole ring in the molecular recognition and shows a binding affinity with DNA higher than RNA. Finally, **Polymer2** was shown to slow down the proliferation of bacteria much more effectively than the free Ag(I) salt.

## 1. Introduction

As all living organisms evolve, it is natural for pathogenic bacteria to develop drug resistance since antimicrobials are commonly used to eradicate them, making them able to counteract by developing resistant strains [1]. In this regard, the excessive and inappropriate use of antibiotics in medicine, animal husbandry, and agriculture accelerates the phenomenon [2]. Managing the increasing number of infections caused by multidrug-resistant bacteria is a serious challenge for modern medicine [1]. It is estimated that in 2019 alone, about 5 million deaths were associated with bacterial antimicrobial resistance [3]. In addition, there are several issues concerning antibiotics already in use, such as their limited bioavailability, poor water solubility, or low stability [4]. The aforementioned facts provide a clear motivation for researchers across the globe to develop new, safe, and more efficient antimicrobial drugs.

So far, imines have found many applications, e.g., as dyes, pigments, or polymer stabilisers. They are convenient intermediates in organic synthesis and the frameworks of many catalysts [5,6]. Schiff base chemistry is shown to be useful in the synthesis of covalent organic frameworks [7] and other nano- and microstructures [8]. Schiff bases are also an important class of ligands in coordination chemistry [9]. Molecules containing the imine group exhibit many promising biological properties, such as antibacterial, antiviral, antifungal, antimalarial, antipyretic, anti-inflammatory, analgesic, antiproliferative and antioxidative activities [5,10,11,12]. Schiff bases are also intermediates in many fundamental bioprocesses [10].

As the problem of microbe resistance to common biocides grows, the need for novel antimicrobial agents has emerged in all its magnitude [13,14]. Aware of the biological potential of Schiff bases and the antimicrobial properties and minor toxicity to human cells of silver ions at low concentrations, we decided to combine those two entities into complexes and explore some of their biological properties. In this regard, similar studies conducted in 2021 by Adeleke et al., who reported the synthesis and biological activities of fifteen Ag(I) quinoline complexes, seemed to corroborate our hypothesis. In fact, all the compounds studied by Adeleke et al. exhibited moderate to excellent antibacterial properties, and two of them were shown to possess significant cytotoxic activity against human cervical cancer (HeLa) cells. Mechanistically, the complexes were also shown to interact with CT-DNA via intercalation [15].

In general, metallotherapeutics have been studied for decades due to the everlasting need for safer and more potent drugs. Pharmaceuticals based on copper, a vital microelement involved in many biological processes, seem a reasonable and potentially less toxic alternative to drugs containing platinum or ruthenium [16,17]. In fact, Cu(II) complexes are a promising group of bioactive agents exhibiting anticancer, antimicrobial, and anti-inflammatory properties, to cite only a few [18]. Cu(I) coordination compounds, despite the oxidative instability of the ions [19], have been studied as potential antitumor [20] and antimicrobial agents as well [16,17]. For instance, in 2022, Villarreal et al. presented the synthesis, structural, and biological studies of a new Cu(I) complex that was shown to be a potential antimalarial drug [21].

Coordination polymers are a particular type of coordination compound, which includes a class of materials consisting of metal ions and organic linkers (ligands) connected together with coordination bonds. Due to their many advantages, e.g., vast diversity and facility of synthesis, they have found numerous applications, especially in the currently developing areas of research, such as catalysis, gas storage and separation, magnetism, nonlinear optics, desalination, etc. If designed appropriately, i.e., using ions of metals endowed with low toxicity, coordination polymers can be used for biomedical purposes, including drug delivery, bioimaging, or biosensing. Some of these materials may exert antimicrobial activities, and their mechanisms of action may vary. For instance, they can be based on ions that have antibacterial properties, such as Ag(I) or Cu(II), whose release follows slow polymer degradation. Another way of eradicating microorganisms is to generate reactive oxygen species or hydrogen peroxide via photocatalysis, which occurs with some metal-organic frameworks based on Zn(II) ions and 2-methylimidazole ligands [22,23,24,25,26].

Herein, we present the synthesis and structural characterisation of five Schiff bases **L1**–**L5** derived from p-xylylenediamine and different aldehydes of heterocyclic moieties [27,28]. The compounds were studied as DNA and RNA ligands using UV-titration experiments. **L1** was used as an organic linker in coordination polymers containing Cu(I) (**Polymer1**) and Ag(I) (**Polymer2**) ions. The bioactivity against a bacterial strain of *Escherichia coli* of the polymers, appropriately Ag(I) and Cu(I) salts, and the ligand itself, were also assessed.

## 2. Results

### 2.1. Design and Synthesis

**Scheme 1:**
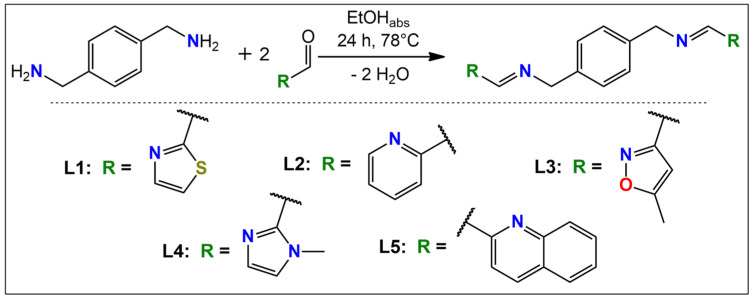
Synthesis of ligands **L1**–**L5** with molecular representations of all derivatives including the respective R moieties.

The five ligands **L1**–**L5** were designed so as to possess two separate N2-donor binding moieties able to coordinate the tetrahedral Ag(I) and Cu(I) ions for their potential use as antibacterial agents. The linker between the two coordinating sites in all ligands was the p-xylyl, while the capping units varied—we used 2-thiophenyl (**L1**), 2-pyridinyl (**L2**), 5-methylisoxazol-3-yl (**L3**), 1-methylimidazol-2-yl (**L4**), and quinoline-2-yl (**L5**, Scheme 1). To gain some insights into the drug-likeness of the proposed molecules, we performed preliminary in silico studies, whose results are summarised in Table 1. A good bioavailability could be achieved with an appropriate balance between solubility and partitioning properties. Thus, in order to achieve good oral drugs, we subjected our compounds to the prediction of the Lipinski “Rule of Five” [29] and other properties for filtering compounds for subsequent synthesis and antimicrobial screening. The most important predictors for the good bioavailability of potential therapeutic agents were the ones given by the above-mentioned Lipinski “Rule of Five.” The rule states that good candidates should have a logP ≤ 5, a molecular weight ≤ 500 g/mol, no more than 10 hydrogen bond acceptors, and a maximum of 5 hydrogen bond donors. The ligands **L1**–**L4** fulfil the Lipinski’s rule [29]. They all also fulfil the rules given by Ghose, Veber, Egan, and Muegge [30,31,32,33]. The only exception was **L5**, which had slightly too high cLogP, according to Lipinski, WLogP and molar refractivity, according to Ghose, and XLogP3, according to Muegge. The calculated polar surface area (tPSA) values of ligands **L1**–**L5** varied from 50.50 to 106.98 Å3, so they were potentially able to cross the membranes.

After this preliminary evaluation, we decided to synthesise all 5 ligands since the coordination of metal ions could improve the solubility and other parameters. All ligands were characterised by ^1^H NMR, ^13^C NMR, ESI-MS, and EA (Figures S1–S10). Moreover, ligands **L1**, **L2**, **L4**, and **L5** were crystallised, and their solid-state structure was confirmed using X-ray (*cf.* Section 2.2). However, only ligand **L1** formed coordination polymers with the initially planned Ag(I) and Cu(I) tetrahedral ions, as revealed by the X-ray diffraction on the single crystals (*cf.* Section 2.2). The polymeric structure of **{[CuL1]PF_6_}_n_** and **{[AgL1]BF_4_}_n_** was also established from the ^1^H NMR spectra (Figures S11 and S12). In the spectra of **Polymer1**, the appropriate peaks were shifted and broader compared to the spectrum of **L1** (Figure S11). It needs to be noted that we took precautions so that the Cu(I) did not oxidise to Cu(II); additionally, the reddish colour of the solution was retained for several weeks. In the case of the Ag(I) polymer, the coordination was evidenced by the shifts of the c, d, and e proton signals compared to the parent spectrum of **L1** (Figure S12). In the literature, there are some reports on the decomposition of Schiff bases upon the addition of metal salt [34], but in the case of our acyclic and macrocyclic Schiff bases, our experimental results indicated that they were highly stable [11,35,36,37].

Ligands **L1–L5** were examined for their interactions with DNA and RNA using UV-Vis titration, while Polymer1 and Polymer2 were tested for their antibacterial activity toward an *E. coli* strain.

### 2.2. Description of Structures

**Figure 1:**
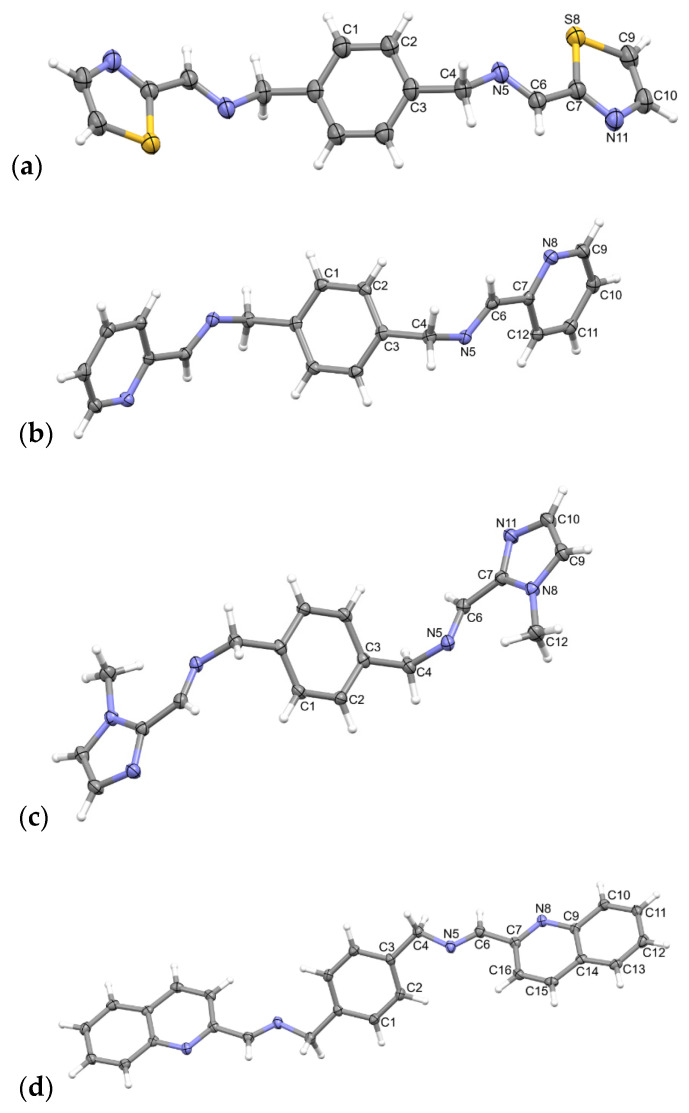
Perspective views of the ligands: (**a**) **L1**, (**b**) **L2**, (**c**) **L4**, and (**d**) **L5**; ellipsoids are drawn at the 50% probability level; hydrogen atoms are shown as spheres of arbitrary radii. The unlabelled part of the molecule is related to the labelled ones using symmetry operations (**a**) −*x*, 1 − *y*, 1 − *z*, (**b**), and (**d**) 1 − *x*, 2 − *y*, −*z*, (**c**) −*x*, 1 − *y*, 2 − *z*.

**Figure 2:**
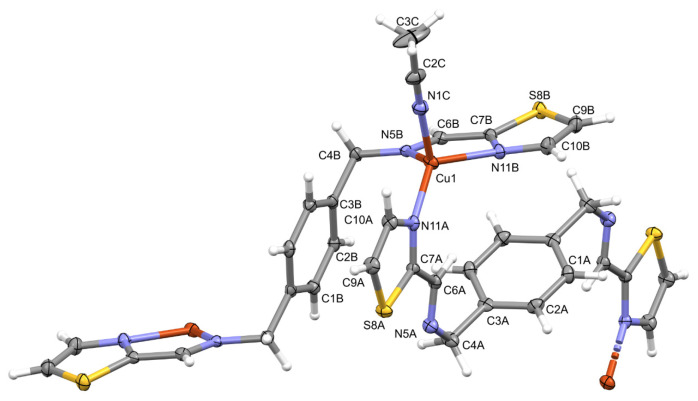
Perspective view of the repetitive fragment of **Polymer1**; ellipsoids are drawn at the 50% probability level; hydrogen atoms are shown as spheres of arbitrary radii. The unlabelled parts of the ligands are related to the labelled ones using symmetry operations (A) 1 − *x*, 1 − *y*, 1 − *z*, (B).

**Figure 3:**
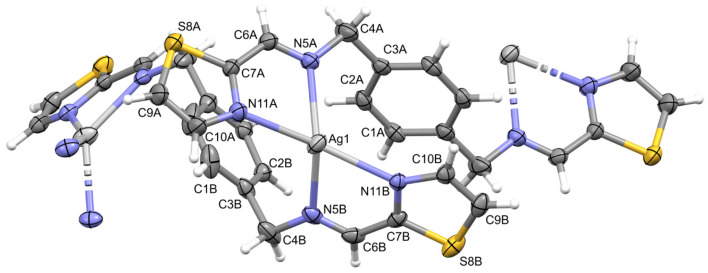
Perspective view of the repetitive fragment of **Polymer2**; ellipsoids are drawn at the 50% probability level; hydrogen atoms are shown as spheres of arbitrary radii. The unlabelled parts of the ligands are related to the labelled ones using symmetry operations (A) 1 − *x*, 2 − *y*, 1 − *z*, (B).

Perspective views of the ligands **L** and cations from the structures **Polymer1** and **Polymer2** are shown in Figure 1, Figure 2 and Figure 3. 

All four ligand molecules in their crystal structures are *C_i_*-symmetrical as they lie across the inversion centres in their respective space groups (**L1** P2_1_/c, all others P-1). As a consequence, the two symmetry-related peripheral rings are exactly parallel. 

Interestingly, in the crystal structures of two cationic complexes, the ligand molecules lie across symmetry elements as well: inversion centres in the case of both ligand molecules in the structure of **Polymer1** and one of the molecules in **Polymer2** and twofold screw for the second molecule in the latter case. As in both structures, the coordination polymers are formed, and the subsequent monomers are related by the inversion centres—two different ones in the case of **L1** and additionally by a twofold screw in **L2**. 

In the Cu complex, **Polymer1**, the Cu ions are four-coordinated by two ligand molecules and by the solvent—the acetonitrile molecule (N_4_) in a distorted tetrahedral environment (Table S1 lists the relevant geometrical data). The two independent ligand molecules display different orientations of the thiazole ring (NCCN disposition is *trans* in molecule A and *cis* in B, cf. Table S1). In consequence, the coordinating potentials of these molecules are different—molecule A acts as a two-dentate ligand (connected to two subsequent Cu ions in the polymeric chain by ring nitrogen atoms only), while molecule B is tetradentate, utilizing all four nitrogen atoms as coordination centres (Figure 2). Therefore, the acetonitrile molecule acts as the fourth coordination place.

**Polymer2** is more typical: in both symmetry-independent ligand molecules, nitrogen atoms are in *cis* disposition (cf. Table S1), and both ligands are tetra-dentate; the Ag ions are coordinated only by the ligand nitrogen atoms, with coordination number 4 and a very severely distorted tetrahedral geometry (Figure 3).

In both polymers, the counterions (PF_6_^−^ and BF_4_^−^) balance the overall charge and lie between the positively charged polymeric chains. It might be safely assumed that the electrostatic interactions between charged species are a main factor for crystal cohesion and the details of crystal architectures. In the structure of **Polymer1**, an additional solvent electron density was found and interpreted as a disordered water molecule.

**Table 1:** Selected physicochemical data for the ligands **L1**–**L5** and the analysis of the drug-likeness of these compounds.

	MW [g/mol]	cLogP	Num. of H-Bond Acceptors	Num. of H-Bond Donors	tPSA [Å]^2^	WLogP	MR	Number of Atoms	Num. of Rotatable Bonds	XlogP3	Number of Rings	Num. of Carbons	Number of Heteroatoms	Lipinski/Ghose/ Veber/Egan/ Muegge Violations
L1	326.44	3.31	4	0	106.98	3.53	93.68	36	6	3.12	3	16	6	0/0/0/0/0
L2	314.38	3.09	4	0	50.50	3.41	97.92	42	6	2.92	3	20	4	0/0/0/0/0
L3	322.36	2.95	6	0	76.78	3.21	92.39	42	6	2.76	3	18	6	0/0/0/0/0
L4	320.39	1.77	4	0	60.36	2.09	96.43	44	6	1.24	3	18	6	0/0/0/0/0
L5	414.50	5.13	4	0	50.50	5.72	132.93	54	6	5.60	5	28	4	1/1/0/0/1
Lipinski [29]	≤500	≤5	≤10	≤5	≤140									
Ghose [30]	160 ≤ MW ≤ 480					−0.4 ≤ Wlog P≤ 5.6	40 ≤ MR ≤ 130	20 ≤ atoms ≤ 70						
Veber [31]					≤140				≤10					
Egan [32]					≤131.6	≤5.88								
Muegge [33]	200 ≤ MW ≤ 500		≤10	≤5	≤150				≤15	−2 ≤ XlogP3 ≤ 5	≤7	>4	> 1	

MW—molecular weight; cLogP—consensus LogP calculated by SwissADME online tool; tPSA—topological polar surface area; WLogP—logP calculated with SwissADME online tool; MR—molar refractivity; XlogP3—logP calculated with atomistic and knowledge-based method calculated using SwissADME online tool. Violations are highlighted in red.

### 2.3. Interactions with Nucleic Acids

In general, the enhanced DNA binding ability of a molecule can be achieved by increasing its planarity with ligands such as bipyridine and phenanthroline. Aromatic compounds, due to their planar structure, have the ability to slide between adjacent nucleic base pairs and induce high destabilization of the DNA double helix (for example with the transition from the B form to the Z form). Previous studies revealed that their planarity promotes intercalative interactions due to π-stacking between the plane of the aromatic rings and DNA base pairs [38,39]. Another important factor is the presence of potential donors and acceptors of H-bonding in the molecules that are directional and specific in the binding with molecules of interest [40].

**Figure 4:**
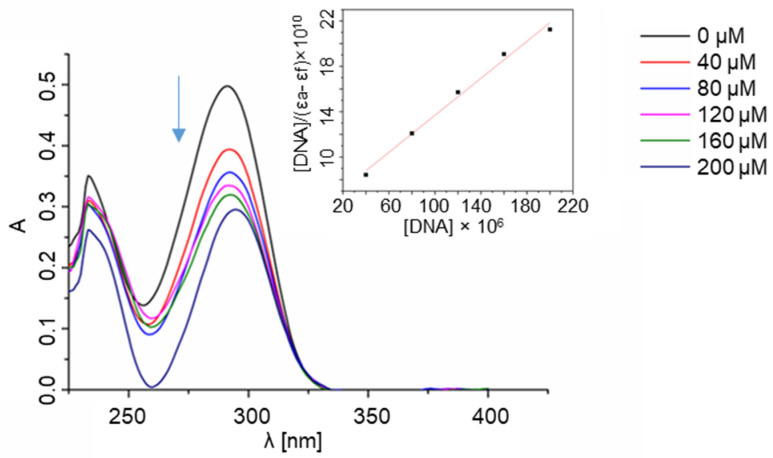
Spectrophotometric titration of **L1** ligand with CT-DNA. Inset: plot of [DNA]/(ε_a_ − ε_f_) versus [DNA]; ▪, experimental data points; solid line, linear fitting of the data.

In order to determine whether ligands interact with the DNA helix, a spectrophotometric titration was performed in our study. The method consists of measuring the UV absorbance of the ligands after each of the subsequent portions of CT-DNA (calf thymus DNA) is added (Figure 4 and Figures S13–S16). The mechanisms of action of many bioactive compounds depend on their binding with DNA; hence, determining the interaction between the molecules and DNA is crucial for understanding, at a molecular level, the origin of their possible therapeutic effects. Chemical compounds may interact with DNA variously—via covalent bonding, intercalation between base pairs, electrostatic interactions with the phosphate-rich backbone, or binding to either minor or major grooves [41,42].

The manner in which a compound interacts with DNA affects the binding’s reversibility, strength, specificity, and cytotoxic effect. When an aromatic compound binds to DNA, its absorbance decreases as the compound is no longer in its free form. In the case of the **L1** ligand, there is a significant decrease in absorbance as the CT-DNA concentration rises, which may indicate the strong interaction with the DNA helix. The bonding stability constant K_b_ of the **L1** ligand equals 1.48 × 10^4^, which is lower than the K_b_ of a standard intercalator ethidium bromide (K_b_ = 1.4 × 10^6^). [14] Due to the conformational lability of the **L1** ligand structure, it is possible that the compound adjusts its conformation to the shape of a major groove with the aid of the thiazole moiety. There are no significant changes in the spectra of the other ligands (Figures S13–S16).

**Figure 5:**
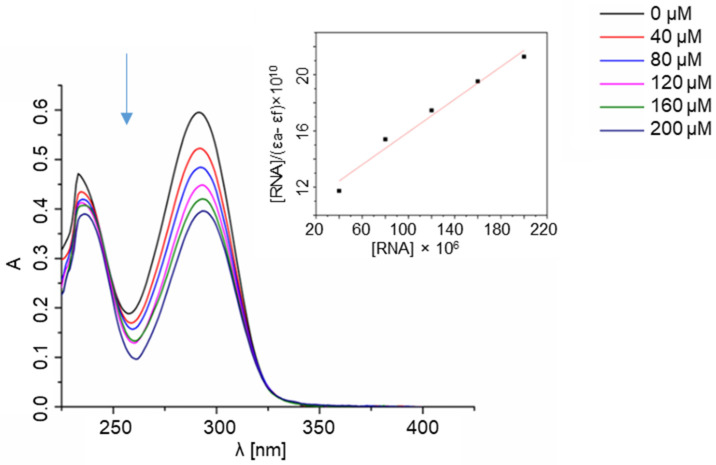
Spectrophotometric titration of **L1** ligand with RNA. Inset: plot of [RNA]/(ε_a_ − ε_f_) versus [RNA]; ▪, experimental data points; solid line, linear fitting of the data.

The next step of the study included an experiment determining the affinity of the ligands to the ribonucleic acid using the RNA from Saccharomyces cerevisiae as a model of RNA (Figure 5 and Figures S17–S20). Given the key structural differences between RNA and DNA, it was expected that the ligands would interact with both types of nucleic acids in non-identical ways. The most important dissimilarity is that RNA is typically single-stranded, while DNA is typically found as a double-strand. RNA molecules play a prominent role in biological processes and evolve as an important target of therapeutic intervention. Molecules that specifically bind to RNA prevent its folding and the formation of RNA-protein complexes. They can also affect cellular functions and have therapeutic potential. Studies conducted over several decades strengthened the role of RNA as a central biomolecule that is considered a structurally and functionally sophisticated biopolymer that participates in key cellular events. For example, RNA can be used to control cell functions via interactions with exogenous ligands and in therapeutics. [43] In accordance with these assumptions, evident differences in the interaction of the ligands with DNA and RNA, stemming from the significant structural dissimilarities of the nucleic targets, were observed by us. 

Per the results of the UV-Vis titration studies, only the ligand **L1** bound to RNA; its bonding stability constant equalled 5.74 × 10^3^. In the case of the **L2**, **L4**, and **L5** ligands, the same phenomenon was not observed. The nonlinear changes in the absorbance in the case of the **L3** ligand might indicate some weak and non-specific electrostatic interactions between the compound and the nucleic acid.

The binding of **L1** to DNA and RNA was also explored computationally by using blind molecular docking with the HDOCK program (see, Experimental section) that revealed the ability of **L1** to interact with DNA (using PDB ID: 1BNA [44], i.e., a model of double-stranded DNA) and RNA (1U2A, a model of RNA from Saccharomyces cerevisiae) [45]. 

**Figure 6:**
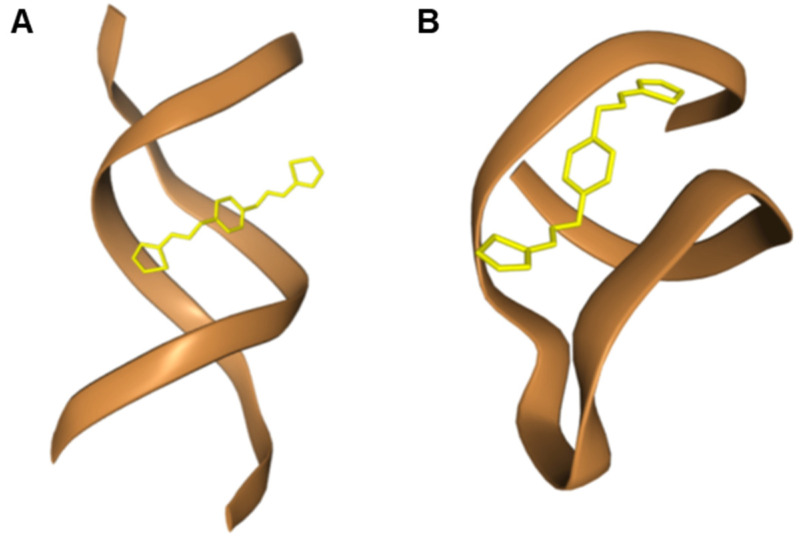
Pose views for the complexes formed by **L1** with DNA (PDB ID: 1BNA, (**A**)) and RNA (PDB ID: 1U2A, (**B**)) as visualized in HDOCK web server (http://hdock.phys.hust.edu.cn/, accessed on 20 September 2022).

**Figure 7:**
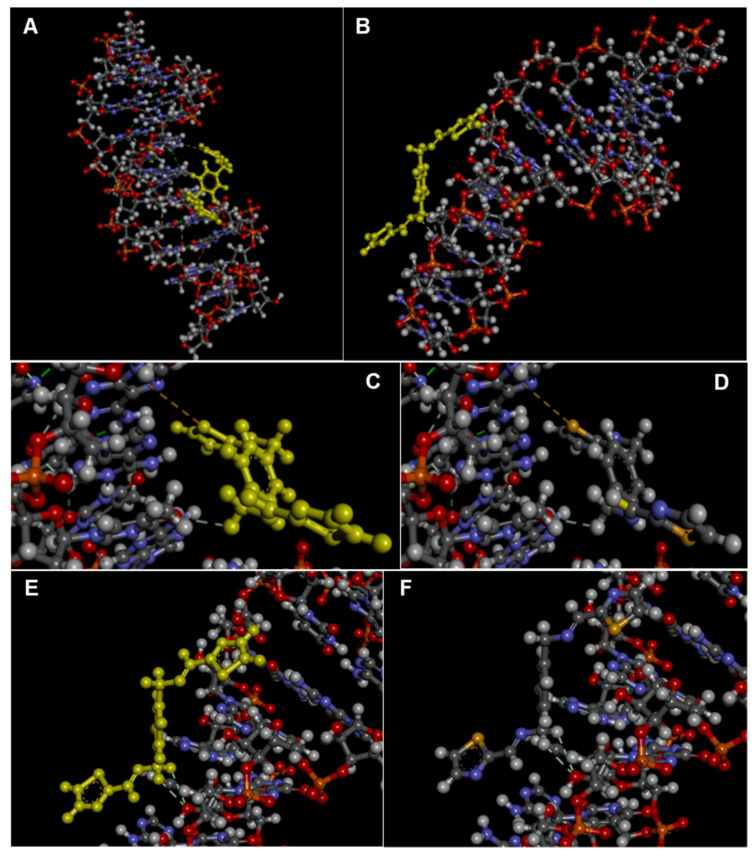
3D view of the top-ranked pose of the complex of **L1** with DNA (**A**) and RNA (**B**) as obtained by docking with the HDOCK server and visualized in Discovery Studio (Dassault Systèmes Corporate, Waltham, MA, USA, v.2021). For the reader’s convenience, the ligand structure is highlighted in yellow. (**C**–**F**) Details of the 3D structures of the complexes of **L1** with DNA (**C**,**D**) and RNA (**E**,**F**) with interactions evidenced as dashed lines as visualized by the software Discovery Studio, showing a sulphur–π stacking interaction between **L1** and DNA (between **L1** S1 and dA5; 5.67 Å) as well as other non-covalent interactions with DNA (carbon–hydrogen bond between **L1** H5 and dT7 O4; 2.95 Å) and RNA (carbon hydrogen bonds **L1** H7-G8 O2′; 2.70 Å and **L1** H8-G8 O2′; 3.00 Å).

In the complex with DNA, no stacking interaction was predicted, but mainly the groove binding of the ligand was evidenced (Figure 6), confirming the hypothesis coming from our experimental findings. Remarkably, the experimental trend of binding affinities was also found in the docking simulation with DNA that formed with **L1** complexes more stable than RNA, as can be deduced by comparing the HDOCK scores in Table 2. This can be explained in terms of stronger interactions involving the thiazole moiety in the case of **L1**-DNA but not **L1**-RNA (Figure 7).

**Table 2:** HDOCK docking results for the top-ranked poses and mean values from the top-1–3 and top-1–10 poses of **L1** complexed with DNA and RNA. The interface residues within 5.0 Å from the ligand in the top-1 complexes are reported in the last column. Note how the energy scores are given by HDOCK as dimensionless, with the most negative values corresponding to the most stable predicted complexes.

	HDOCK Score Top-1 Ranked Pose	HDOCK Score (Top-1–3 Poses) ± SD	HDOCK Score (Top-1–10 Poses) ± SD	Interface Residues
DNA *	−70.22	−69.45 ± 0.69	−66.76 ± 2.37	dA5, dA6, dT7, dT19
RNA **	−62.25	−61.57 ± 0.64	−57.69 ± 3.67	U7, G8, U9, C12, A13, C15, U16

* model DNA PDB ID: 1BNA. ** model RNA PDB ID: 1U2A.

### 2.4. Effect of Selected Compounds on Bacterial Proliferation

**Figure 8:**
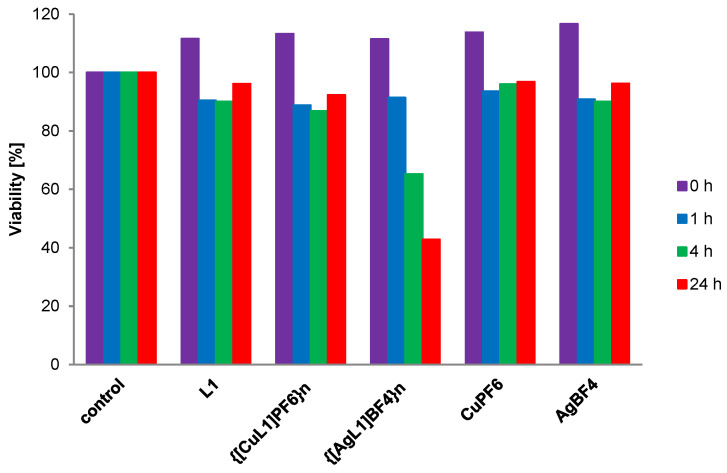
The effect of **L1**, **Polymers 1** and **2** and the reference salts (all at a 100 µM concentration) on the proliferation of *Escherichia coli* bacteria as a function of time.

The effect of ligand **L1**, **Polymer1 {[CuL1]PF_6_}_n_**, and **Polymer2 {[AgL1]BF_4_}_n_** on bacterial growth was tested, and, in particular, the effect of the compounds on the change in the number of bacteria after times of 1, 4, and 24 h was investigated, as shown in Figure 8. 

**Figure 9:**
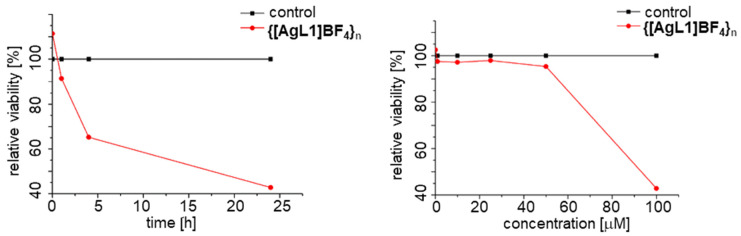
(**left**) The effect of **Polymer2 {[AgL1]BF_4_}_n_** on the number of bacteria as a function of time. (**right**) The effect of **Polymer2 {[AgL1]BF_4_}_n_** on the number of bacteria depending on the concentration of the compound in the solution.

The highest activity against *E. coli* bacteria was shown by the **Polymer2 {[AgL1]BF_4_}_n_**, whose MIC50 (minimum inhibitory concentration of a compound that inhibits the growth of microorganisms by 50%) was found to be 92 μM after 24 h (Figure 9). At the same time, the activity of this complex highly exceeded that of silver salt (AgBF_4_) used as a control, ruling out the exclusive influence of the silver ion (Figure 9, left). From a biomedical perspective, the **Polymer2** containing tetradentate ligand **L1** in *cis* disposition, coordinating Ag(I) in a tetrahedral fashion, was shown to slow down the proliferation of bacteria much more effectively than the free Ag(I) salt. In this respect, we hypothesize that this could be an effect of the increased lipophilicity of the material or of a prolonged “metal-drug” release from the coordination polymer. Increased activity of complexes might be explained by the Tweedy chelation theory: it is observed that the positive charge in the complex is partially reduced due to the overlap of the ligand orbital. Further, it increases both the delocalization of π-electrons over the whole chelate structure and the lipophilic character of the complex, which enhances the penetration of the compound into the lipid layer of the bacterial cell membranes and blocks the metal binding sites in enzymes. [46] Previous preliminary results suggest that the bactericidal mechanism of Ag(I) ions occurs via DNA condensation and that the diminished replication abilities are a reaction against the denaturation process. Moreover, silver ions may interact with thiol groups in proteins, which induces the deterioration of cellular functions [14]. At the same time, ligand **L1** and the **Polymer1 {[CuL1]PF_6_}_n_** showed only moderate strain-directed activity, which was comparable to that of silver and copper salts.

## 3. Conclusions

In conclusion, five new ligands were synthesized, characterized and investigated in biomolecular interaction assays, revealing the ability of **L1** to bind DNA and RNA, as evidenced by UV titration. The interaction with DNA was mediated by interactions with one of the two thiazole moieties, while the **L1**-RNA binding was driven by weaker forces involving atoms out of the thiazole ring, as suggested by the molecular docking simulation.

Remarkably, **L1** was able to form coordination polymers with Ag(I) and Cu(I) that were crystallized and described in a detailed form in our work. **Polymer2** containing Ag(I) was shown to slow down the proliferation of bacteria more effectively than the free Ag(I) salt, while **L1** and **Polymer1** containing Cu(I) showed only moderate activity. 

Overall, all the reported findings of this work concur to depict **L1** as a thiazole-based heteroaromatic derivative with important characteristics as a binder of nucleic acids (as we showed with the two DNA and RNA models) and as a ligand of metals endowed with antimicrobial properties, as we demonstrated in particular in the case of the **L1**-based polymer containing Ag(I). Therefore, new studies involving both the synthesis of new modified **L1** analogues and biophysical/biological assays with other biomolecular targets are clearly desirable. 

## 4. Experimental

### 4.1. Materials and Methods

CT-DNA was purchased from Merck (Darmstadt, Germany), while the baker’s yeast RNA from S. cerevisiae was Alfa Aesar (Heysham, UK). All reagents (Merck, Darmstadt, Germany) and substrates were used without further purification. ESI mass spectra for MeCN solutions ~10^−4^ M were measured using a Waters Co. Micromass ZQ spectrometer (Milford, CT, USA) and QTOF type mass spectrometer Impact HD, Bruker (Billerica, MA, USA). NMR spectra were run on a Varian Gemini 300 MHz spectrometer (Oxford, UK) and were calibrated against the residual protonated solvent signals with chemical shifts represented in ppm. Microanalyses were performed using a VarioEL III CHN element analyzer (Thermo Scientific, Waltham, MA, USA).

CT-DNA and RNA were dissolved in a PBS buffer, pH = 7.4, prior to use. The CT-DNA solution gave a ratio of UV absorbance of 1.82:1 at 260 and 280 nm, indicating that the CT-DNA sample was sufficiently free from protein [47]. CT-DNA and RNA concentrations per nucleotide were determined from the UV absorbance at 260 nm using the extinction coefficient ε_260_ = 6600 dm^3^·mol^−1^·cm^−1^ and ε_260_ = 7800 dm^3^·mol^−1^·cm^−1^, respectively [48]. Electronic absorption spectra were performed on UV–Vis JASCO V-770 equipped with a Peltier Thermo Cell Holder (water) PAC-743R (Jasco International Co., Tokyo, Japan).

It needs to be emphasized that compounds are stable in this medium for several weeks (after this time, some precipitate starts to occur).

### 4.2. Synthesis of Ligands L1–L5

The ligands were synthesized according to the procedure depicted in Scheme 1.

**L1—C_16_H_14_N_4_S_2_**

The 2-thiazolecarboxaldehyde (343.94 mg, 3.03 mmol) was dissolved in 6 mL absolute EtOH. Then p-xylylenediamine (200 mg, 1.46 mmol) was added. The reaction was carried out for 24 h under an argon atmosphere at 78 °C. The product was precipitated with diethyl ether and was filtered, washed with cold absolute EtOH, and dried under reduced pressure for 5 h. A yellow product was obtained with a 72.0% yield. Single crystals suitable for X-ray diffraction analysis were formed by a slow diffusion of diisopropyl ether into the sample solution in acetonitrile at 4 °C over a period of 6–8 weeks. 

Anal. Calcd. for C_16_H_14_N_4_S_2_ (326.43 g mol^−1^): C, 58.87; H, 4.32; N, 17.16; S, 19.65. Found C, 58.68; H, 4.57; N, 17.22; S, 19.47%. 

^1^H NMR (ACN-d_3_, 300 MHz): 8.66 (s, 2H), 7.93 (d,2H), 7.56 (d, 2H), 7.43 (s, 4H), 4.82 (s, 4H). 

^13^C NMR (DMSO-d_6_, 75 MHz): 166.50, 156.03, 144.23, 137.43, 128.37, 122.97, 62.93.

ESI-MS (+)(%) [NaL1]^+^ 349 (100), (%) [HL1]^+^ 327 (25).

**L2—C_20_H_18_N_4_**

The pyridine-2-carboxaldehyde (293.2 µL, 3.1 mmol) was dissolved in 8 mL absolute EtOH. Then p-xylylenediamine (200.00 mg, 1.46 mmol) was added. The reaction was carried out for 24 h under an argon atmosphere at 78 °C. The product was precipitated with diethyl ether and was filtered, washed with cold absolute EtOH and dried under reduced pressure for 5 h. A white product was obtained with a 70.0% yield. Single crystals suitable for X-ray diffraction analysis were formed by a slow diffusion of diisopropyl ether into the sample solution in acetonitrile at 4 °C over a period of 6-8 weeks. 

Anal. Calcd. for C_20_H_18_N_4_ (314.38 g mol^−1^): C, 76.41; H, 5.77; N, 17.82. Found C, 76.66; H, 5.26; N, 17.93%.

^1^H NMR (DMSO-d_6_, 300 MHz): 8.66 (d, 2H), 8.50 (s,2H), 8.00 (d, 2H), 7.87 (t, 2H), 7.48 (t, 2H), 7.35 (s, 4H), 4.84 (s, 4H). 

^13^C NMR (DMSO-d_6_, 75 MHz): 162.60, 154.09, 149.41, 137.76, 136.92, 128.23, 125.23, 120.50, 63.55. 

ESI-MS (+) (%) [HL2]^+^ 315 (100), (%) [NaL2]^+^ 337 (55).

**L3—C_18_H_18_N_4_O_2_**

The 5-methylisoxazole-3-carboxaldehyde (342.31 mg, 3.1 mmol) was dissolved in 8 mL absolute EtOH. Then p-xylylenediamine (200.00 mg, 1.46 mmol) was added. The reaction was carried out for 24 h under an argon atmosphere at 78 °C. The product was precipitated with diethyl ether and was filtered, washed with cold absolute EtOH and dried under reduced pressure for 5 h. A white product was obtained with a 68.0% yield.

Anal. Calcd. for C18H18N4O2 (322.37g mol^−1^): C, 67.07; H, 5.63; N, 17.38. Found C, 67.07; H, 5.63%; N, 17.38%.

^1^H NMR (DMSO-d_6_, 300 MHz): 8.55 (s, 2H), 7.31 (s, 4H), 6.53 (s, 2H), 4.83 (s, 4H), 2.44 (s, 6H). 

^13^C NMR (DMSO-d_6_, 75 MHz): 170.45, 161.86, 152.77, 137.37, 128.25, 99.32, 63.75, 11.76. 

ESI-MS (+) (%) [HL3]^+^ 323 (75), (%) [NaL3]^+^ 345 (10).

**L4**—**C_18_H_20_N_6_**

The 1-methyl-2-imidazolecarboxaldehyde (339.57 mg, 3.1 mmol) was dissolved in 6 mL absolute EtOH. Then p-xylylenediamine (200.00 mg, 1.46 mmol) was added. The reaction was carried out for 24 h under an argon atmosphere at 78 °C. The product was precipitated with diethyl ether and was filtered, washed with cold absolute EtOH and dried under reduced pressure for 5 h. A white product was obtained with a 68.0% yield. Single crystals suitable for X-ray diffraction analysis were formed by a slow diffusion of diisopropyl ether into the sample solution in acetonitrile at 4 °C over a period of 6-8 weeks. 

Anal. Calcd. for C_18_H_20_N_6_ (320.40 g mol^−1^): C, 67.48; H, 6.29; N, 26.23. Found C, 67.54; H, 6.379; N, 26.88%.

^1^H NMR (ACN-d_3_, 300 MHz): 8.37 (s, 2H), 7.33 (s, 4H), 7.10 (d, 2H), 7.05 (d, 2H), 4.75 (s, 4H), 3.95 (s, 6H).

^13^C NMR (ACN-d_3_, 75 MHz): 33.20, 63.02, 123.80, 126.42, 127.29, 136.91, 141.49, 152.57.

ESI-MS (+) (%) [HL4]^+^ 321 (100), (%) [NaL4]^+^ 343 (10).

**L5—C_28_H_22_N_6_**

The quinoline-2-carboxaldehyde (600 mg, 3.8 mmol) was dissolved into 10 mL absolute EtOH. Then p-xylylenediamine (258 mg, 2.098 mmol) was added. The reaction was carried out for 24 h under an argon atmosphere at 78 °C. The product was precipitated with diethyl ether and was filtered, washed with cold absolute EtOH and dried under reduced pressure for 5 h. A white product was obtained with a 64.0% yield. Single crystals suitable for X-ray diffraction analysis were formed by a slow diffusion of diisopropyl ether into the sample solution in acetonitrile at 4 °C over a period of 6–8 weeks.

Anal.: Calcd. for C_28_H_22_N_6_ (414.50 g mol^−1^): C, 81.13; H, 5.35; N, 13.52, Found C, 81.17; H, 5.31; N, 13.64%.

^1^H NMR (DMSO-d_6_, 300 MHz): 8.66 (s, 2H); 8.42 (d, 2H); 8.12 (d, 1H); 8.05 (d, 2H); 8.02 (d, 2H); 7.81 (t, 2H); 7.66 (t, 2H); 7.40 (s, 2H); 4.82 (s, 4H).

^13^C NMR (DMSO-d_6_, 75 MHz) 163.32, 154.89, 147.66, 138.16, 137.37, 130.58, 129.59, 128.86, 128.86, 128.82, 128.51, 128.13, 118.33, 63.97. 

ESI-MS (+) (%) [HL5]^+^ 415 (100).

### 4.3. Synthesis of the Coordination Polymers 

**Polymer1—{[CuL1]PF_6_}_n_**

The L1 ligand (50.00 mg, 153.1 µmol) was dissolved in acetonitrile (15 mL), and then CuPF_6_ salt (57.10 mg, 153.0 µmol) was added. Argon was passed through the solution to remove air to prevent the oxidation of copper ions. The reaction was carried out for 24 h at room temperature. The solution was then reduced to a volume of about 3 mL, and the precipitate was obtained by the addition of the diethyl ether. The orange product was filtered under reduced pressure and dried in a vacuum desiccator, with a yield of 58%. By the method of slow diffusion in the acetonitrile/diisopropyl ether solvent system, a monocrystal suitable for diffractometric measurements was obtained. 

ESI-MS (%): [L1Na]^+^ 349 (100), [Cu(L1)_2_]^+^ 715 (65), [CuL1]^+^ 389 (50), [HL1]^+^ 327 (40).

^1^H NMR (300 MHz, ACN-d3): 8.60 (broad peak, 2H), 8.20-7.60 (broad peak, 4H), 7.33 (s, 4H), 4.85 (s, 4H).

**Polymer2—[AgL1]BF_4_}_n_**

The L1 ligand (50.00 mg, 153.1 µmol) was dissolved in acetonitrile (15 mL), and then AgBF_4_ salt (29.80 mg, 153.0 µmol) was added. The reaction was carried out for 24 h at room temperature, protecting the reaction flask from the sunlight. The solution was then reduced to a volume of about 5 mL, and a white precipitate was obtained with diethyl ether. The off-white product was filtrated under reduced pressure and dried in a vacuum desiccator with a yield of 35%. By the method of slow diffusion of diethyl ether into the acetonitrile solution of the complex, monocrystals suitable for X-ray structural analysis were obtained. 

^1^H NMR (300 MHz, ACN-d3): 8.57 (s, 2H), 7.90 (d, 2H), 7.67 (d, 2H), 7.27 (s, 4H), 4.78 (s, 4H).

ESI-MS (−)(%) [AgL1(BF_4_)_2_]^−^ 606 (10).

### 4.4. X-ray Crystallography

Diffraction data were collected using the ω-scan technique for **L1** and **Polymer2** at 130(1) K on a Rigaku SuperNova four-circle diffractometer with an Atlas CCD detector, equipped with Nova microfocus CuK_α_ radiation source (λ = 1.54178 Å), and for **L2**, **L4**, **L5**, and **Polymer1** at 100(1) K, on a Rigaku XCalibur four-circle diffractometer with an Eos CCD detector, with a graphite-monochromatised MoK_α_ radiation source (λ = 0.71073 Å). The data were corrected for Lorentz-polarization as well as for absorption effects [49]. The structures were solved with SHELXT [50] and refined with the full-matrix least-squares procedure on F^2^ by SHELXL-2013 [51]. All non-hydrogen atoms were refined anisotropically, and hydrogen atoms were placed in idealised positions and refined as ‘riding models’ with isotropic displacement parameters set at 1.2 (1.5 for methyl and hydroxyl groups) times U_eq_ of appropriate carrier atoms. In the structure of **Polymer1**, the relatively high residual density far from the rest of the structure was interpreted as a disordered water molecule, with s.o.f’s set at 1/3 and 2/3 on the basis of keeping similar displacement parameters. The relevant crystallographic data, together with the details of structure refinement, are listed in Table 3.

### 4.5. In Silico Pharmacokinetic Property Studies

The SMILES codes for compounds (Simplified Molecular Input Line Entry System) were obtained and applied to the calculations using the SwissADME [52] (http://www.swissadme.ch, accessed on 20 November 2022): the molecular weights (MW), the logP values (octanol-water partition coefficient), in six variants (ILOGP, XLOGP3, WLOGP, MLOGP, SILICOS-IT and consensus LogP, cLogP, which was an average of five mentioned predictions), tPSAs (topological polar surface area), number of hydrogen-bond acceptors and donors, number of atoms, rotatable bonds, ring, carbon and heteroatoms. The cLogP values were obtained using the SwissADME web service [47], together with ILOGP, obtained with the in-house physics-based method implemented by Daina et al. [53]; XLOGP3 values, predicted with the atomistic and knowledge-based method calculated using the XLOGP program, version 3.2.2, courtesy of CCBG, Shanghai Institute of Organic Chemistry; WLOGPs, obtained with the atomic method implemented by Wildman SA and Crippen GM [54]; MLOGPs, calculated with the topological method implemented by Moriguchi et al. [55,56] and Lipinski et al. [57]; SILICOS-IT scores, obtained with the hybrid fragmental/topological method calculated using the FILTER-IT program, version 1.0.2, courtesy of SILICOS-IT (http://ww1.silicos-it.com/, accessed on 20 November 2022); other parameters not mentioned here using the SwissADME web service [52]. The tPSA value was calculated according to Ertl et al. [58]

SMILES codes:

**L1**: C(\N=C\C1=NC=CS1)C1=CC=C(C\N=C\C2=NC=CS2)C=C1

**L2**: C(\N=C\C1=CC=CC=N1)C1=CC=C(C\N=C\C2=NC=CC=C2)C=C1 

**L3**: CC1=CC(\C=N\CC2=CC=C(C\N=C\C3=NOC(C)=C3)C=C2)=NO1

**L4**: CN1C=CN=C1\C=N\CC1=CC=C(C\N=C\C2=NC=CN2C)C=C1

**L5**: C(\N=C\C1=NC2=C(C=CC=C2)C=C1)C1=CC=C(C\N=C\C2=NC3=C(C=CC=C3)C=C2)C=C1

### 4.6. Spectrophotometric Titration of the Ligands with Nucleic Acids

#### 4.6.1. Ligand–DNA Interactions

Spectrophotometric titrations of the ligands with CT-DNA were performed as follows. Starting solutions of the ligands in DMSO (c = 2 mM) were first prepared. A PBS buffer with pH = 7.4 was used in the measurements, with 2.45 mL of such buffer and 50 μL of the ligand solution being placed in a quartz cuvette (with dimensions 1 cm × 1 cm). Subsequently, the absorption spectrum was measured (at c_CT-DNA_ = 0 μM). Before each subsequent measurement, a portion of CT-DNA was added, increasing its concentration by 40 μM each time until the final concentration of CT-DNA was reached (c_CT-DNA_ = 200 μM). Six measurements were conducted for each of the ligands individually. The baseline was measured before every measurement for each of the CT-DNA concentrations in the buffer. The ligand–DNA binding constant K_b_ was calculated for the **L1** ligand according to the equation:

[DNA]/(ε_a_ − ε_f_) = [DNA]/(ε_b_ − ε_f_) + 1/K_b_ (ε_b_ − ε_f_),

where [DNA] is the concentration of CT-DNA in base pairs, ε_a_ is the observed extinction coefficient, ε_f_ is the extinction coefficient of the compound in its free form, and ε_b_ is the extinction coefficient of the compound fully bound to CT-DNA [59].

#### 4.6.2. Ligand-RNA Interactions

Spectrophotometric titration of the ligands with RNA was also performed. Similarly to DNA, starting solutions of the ligands in DMSO (c = 2 mM) were prepared, and the PBS buffer with pH = 7.4 was used in the measurements. An amount of 2.45 mL of the buffer and 50 μL of the ligand solution were placed in a quartz cuvette (with dimensions 1 cm × 1 cm), and then the absorption spectrum was measured (at c_RNA_ = 0 μM). Before each subsequent measurement, a portion of RNA was added, increasing its concentration by 40 μM until the final concentration of RNA was reached (c_RNA_ = 200 μM). Six measurements were conducted for each of the ligands individually. The baseline was measured before every measurement for each of the RNA concentrations in the buffer. The ligand–RNA binding constant K_b_ was calculated for the **L1** ligand according to the equation:

[RNA]/(ε_a_ − ε_f_) = [RNA]/(ε_b_ − ε_f_) + 1/K_b_ (ε_b_ − ε_f_),

where [RNA] is the concentration of RNA in base pairs, ε_a_ is the observed extinction coefficient, ε_f_ is the extinction coefficient of the compound in its free form, and ε_b_ is the extinction coefficient of the compound fully bound to RNA [59].

### 4.7. Effect of Compounds on Bacterial Proliferation

An antimicrobial assessment was conducted to determine the effect of ligand **L1** and its complexes **{[CuL1]PF_6_}_n_** and **{[AgL1]BF_4_}_n_** on the proliferation of a bacterial strain of *Escherichia coli*. Stock solutions of the compounds were prepared with concentrations of c = 10 mM, c = 5 mM, and c = 2.5 mM. To the sets of liquid cultures (5 mL), 50 µL each of the previously prepared compound solutions was added. After 100× dilution, the concentrations in the test samples was 100 µM, 50 µM, and 25 µM, respectively. The OD (optical density) was measured both before and after incubation periods of 1 h, 4 h, and 24 h. The initial OD for the bacteria was 0.38. Measurements were also made for the corresponding metal salts used in the synthesis. Bacteria in a medium (without any of the tested compounds) were the control sample.

### 4.8. Molecular Docking Studies

We performed molecular docking simulations [60,61,62,63] with the HDOCK server (http://hdock.phys.hust.edu.cn, accessed on 20 September 2022) [64,65], suitable for both macromolecule-to-macromolecule [64] and macromolecules-to-small molecule [66] rigid dockings, using default parameters. The PDB entries 1BNA [44], relative to the DNA dodecamer d(CpGpCpGpApApTpTpCpGpCpG), and 1U2A, relative to the stem-loop IIa from yeast U2 small nuclear RNA [45], were used as models of DNA and RNA, respectively, for our blind dockings. They were furnished to HDOCK as targets, while the 3D structure of **L1** saved as a .pdb file was uploaded into the server as the ligand. Thanks to the iterative knowledge-based scoring function ITScore-PP, the HDOCK server ranked the top ten poses obtained after the dockings. The energy score (HDOCK score) values given by the program, and predicted by ITScore-PP, were dimensionless, with larger negative numbers being linked to higher affinity interactions between the interacting ligand and the target macromolecule, which was also reported to correlate well to experimental binding affinities showing a correlation coefficient of R = 0.71 [67]. More details on the procedures for the docking and the HDOCK docking server itself are available at http://hdock.phys.hust.edu.cn (accessed on 20 September 2022). We analysed the top-ranked pose (Top-1) for the complexes predicted using HDOCK according to the energy scores provided by the program, as explained in the Results and discussion section.

## Figures and Tables

**Table 3:** Crystal data, data collection and structure refinement.

Compound	L1	L2	L4
Formula	C_16_H_14_N_4_S_2_	C_20_H_18_N_4_	C_18_H_20_N_6_
Formula weight	326.43 /c	314.38 /c	320.40 /c
Crystal system	monoclinic	Triclinic	triclinic
Space group	P2_1_/c	P-1	P-1
a (Å)	9.0685 (4)	4.4796 (3)	6.0318 (5)
b (Å)	7.5193 (2)	9.9845 (12)	7.7536 (7)
c (Å)	11.6105 (4)	10.2941 (11)	9.1295 (10)
α (°)	90	61.700 (11)	79.190 (8)
β (°)	108.245 (4)	84.554 (7)	80.956 (8)
γ (°)	90	81.318 (8)	78.176 (7)
V (Å^3^)	751.90 (5)	400.60 (8)	407.35 (7)
Z	2	1	1
D_x_(g cm^−3^)	1.442	1.303	1.306
F(000)	340	166	170
μ(mm^−1^)	3.213	0.080	0.083
Reflections:			
collected	2967	2434	2598
unique (R_int_)	1526 (0.0318)	1547 (0.0063)	1565 (0.0144)
with I > 2σ(I)	1395	1441	1344
R(F) [I > 2σ(I)]	0.0539	0.0317	0.0429
wR(F^2^) [I > 2σ(I)]	0.1397	0.0783	0.1128
R(F) [all data]	0.0567	0.0338	0.0509
wR(F^2^) [all data]	0.1429	0.0800	0.1184
Goodness of fit	1.037	1.068	1.041
max/min Δρ (e·Å^−3^)	0.62/−0.49	0.20/−0.18	0.27/−0.22
CCDC number	2209859	2209860	2209861
**Compound**	**L5**	**Polymer1**	**Polymer2**
Formula	C_28_H_22_N_4_	C_36_H_34_Cu_2_N_10_S_4_· 2PF_6_·2H_2_O	C_32_H_28_Ag_2_N_8_S_4_· 2BF_4_
Formula weight	414.49 /c	1188.02 /c	1042.22 /c
Crystal system	triclinic	triclinic	monoclinic
Space group	P-1	P-1	C2/c
a (Å)	5.9704 (6)	8.6731 (2)	17.21206 (16)
b (Å)	9.3939 (11)	12.2325 (3)	11.57724 (11)
c (Å)	10.3962 (11)	12.4167 (3)	18.9418 (2)
α (°)	102.733 (10)	105.634 (2)	90
β (°)	101.646 (9)	105.729 (2)	98.0626 (9)
γ (°)	107.981 (10)	98.175 (2)	90
V(Å^3^)	517.56 (10)	1187.71 (5)	3737.19 (6)
Z	1	1	4
D_x_(g cm^−3^)	1.330	1.661	1.852
F(000)	218	600	2064
μ(mm^−1^)	0.080	1.231	11.198
Reflections:			
collected	3561	23396	31659
unique (R_int_)	2066 (0.0196)	4907 (0.0152)	3908 (0.0576)
with I > 2σ(I)	1603	4768	3819
R(F) [I > 2σ(I)]	0.0472	0.0325	0.0464
wR(F^2^) [I > 2σ(I)]	0.1007	0.0755	0.1502
R(F) [all data]	0.0633	0.0333	0.0469
wR(F^2^) [all data]	0.1099	0.0759	0.1510
Goodness of fit	1.059	1.015	1.084
max/min Δρ (e·Å^−3^)	0.19/−0.22	0.65/−0.50	1.36/−1.35
CCDC number	2209862	2209863	2209864

## Data Availability

Not applicable.

## Supplementary Materials

The following supporting information can be downloaded at: https://www.mdpi.com/article/10.3390/molecules28010400/s1, Figure S1 ^1^H NMR spectrum of ligand **L1** in ACN-d3.; Figure S2 ^13^C NMR spectrum of ligand **L1** in DMSO-d6.; Figure S3 ^1^H NMR spectrum of ligand **L2** in DMSO-d6.; Figure S4 ^13^C NMR spectrum of ligand **L2** in DMSO-d6.; Figure S5 ^1^H NMR spectrum of ligand **L3** in DMSO-d6.; Figure S6 ^13^C NMR spectrum of ligand **L3** in DMSO-d6.; Figure S7 ^1^H NMR spectrum of ligand **L4** in ACN-d3.; Figure S8 ^13^C NMR spectrum of ligand **L4** in ACN-d3.; Figure S9 ^1^H NMR spectrum of ligand **L5** in DMSO-d6.; Figure S10 ^13^C NMR spectrum of ligand **L5** in DMSO.; Figure S11 Superimposed ^1^H NMR spectra of **L1** and coordination polymer {[Cu**L1**]PF_6_}_n_ in ACN-d3; Figure S12 Superimposed ^1^H NMR spectra of **L1** and coordination polymer {[Ag**L1**]BF_4_}_n_ in ACN-d3.; Figure S13 Spectrophotometric titration of **L2** ligand with CT-DNA.; Figure S14 Spectrophotometric titration of **L3** ligand with CT-DNA.; Figure S15 Spectrophotometric titration of **L4** ligand with CT-DNA.; Figure S16 Spectrophotometric titration of **L5** ligand with CT-DNA.; Figure S17 Spectrophotometric titration of **L2** ligand with RNA.; Figure S18 Spectrophotometric titration of **L3** ligand with RNA.; Figure S19 Spectrophotometric titration of **L4** ligand with RNA. Figure S20 Spectrophotometric titration of **L5** ligand with RNA. Table S1 Relevant geometrical parameters (Å, °) with su’s in parentheses. i denotes the symmetry operation 1−x, 1−y, 1−z. X and Y are mean planes of the phenyl and thiazole rings in the ligand molecules.

## Sample Availability

Samples of all the compounds are available from the authors.
