# Supplementary material for: New N4-Donor Ligands as Supramolecular Guests for DNA and RNA: Synthesis, Structural Characterization, In Silico, Spectrophotometric and Antimicrobial Studies
Source: Molecules. 2023 Jan 3;28(1):400. doi: 10.3390/molecules28010400 (PMC9823393; doi:10.3390/molecules28010400)
Supplement: Supplementary file 1 [file molecules-28-00400-s001.zip › molecules-2111859-supplementary.pdf]

Supplementary materials

Table S 1 Relevant geometrical parameters (Å, °) with su's in parentheses. i denotes the symmetry operation 1-x,1-y,1-z. X and Y are mean planes of the phenyl and thiazole rings in the ligand molecules....2
Figure S 1 ^{1}H NMR spectrum of ligand **L1** in ACN-d3. 3
Figure S 2 ^{13}C NMR spectrum of ligand **L1** in DMSO-d6. 3
Figure S 3 ^{1}H NMR spectrum of ligand **L2** in DMSO-d6. 4
Figure S 4 ^{13}C NMR spectrum of ligand **L2** in DMSO-d6. 4
Figure S 5 ^{1}H NMR spectrum of ligand **L3** in DMSO-d6. 5
Figure S 6 ^{13}C NMR spectrum of ligand **L3** in DMSO-d6. 5
Figure S 7 ^{1}H NMR spectrum of ligand **L4** in ACN-d3. 6
Figure S 8 ^{13}C NMR spectrum of ligand **L4** in ACN-d3.... 6
Figure S 9 ^{1}H NMR spectrum of ligand **L5** in DMSO-d6. 7
Figure S 10 ^{13}C NMR spectrum of ligand **L5** in DMSO.... 7
Figure S 11 Superimposed ^{1}H NMR spectra of **L1** and coordination polymer $\{[Cu\mathbf{L1}]PF_6\}_n$ in ACN-d3. .. 8
Figure S 12 Superimposed ^{1}H NMR spectra of **L1** and coordination polymer $\{[Ag\mathbf{L1}]BF_4\}_n$ in ACN-d3. .. 8
Figure S 13 Spectrophotometric titration of **L2** ligand with CT-DNA. 9
Figure S 14 Spectrophotometric titration of **L3** ligand with CT-DNA. 9
Figure S 15 Spectrophotometric titration of **L4** ligand with CT-DNA. 9
Figure S 16 Spectrophotometric titration of **L5** ligand with CT-DNA. 10
Figure S 17 Spectrophotometric titration of **L2** ligand with RNA. 10
Figure S 18 Spectrophotometric titration of **L3** ligand with RNA. 10
Figure S 19 Spectrophotometric titration of **L4** ligand with RNA. 11
Figure S 20 Spectrophotometric titration of **L5** ligand with RNA. 11

Table S 1 Relevant geometrical parameters (Å, °) with su's in parentheses. [i] denotes the symmetry operation 1-x,1-y,1-z. X and Y are mean planes of the phenyl and thiazole rings in the ligand molecules.

**Polymer1**		**Polymer2**	
Cu1-N11A	1.997(2)	Ag1-N5A	2.305(4)
Cu1-N5B	2.060(2)	Ag1-N11A	2.514(3)
Cu1-N11B	2.116(2)	Ag1-N5B	2.335(4)
Cu1-N1C	1.969(2)	Ag1-N11B	2.428(3)
N11A-Cu1-N5B	120.58(8)	N5A-Ag1-N11A	70.90(11)
N11A-Cu1-N11B	123.67(8)	N5A-Ag1-N5B	133.55(12)
N11A-Cu1-N1C	106.45(9)	N5A-Ag1-N11B	119.34(10)
N5B-Cu1-N11B	80.47(8)	N11A-Ag1-N5B	117.70(12)
N5B-Cu1-N1C	115.62(9)	N11A-Ag1-N11B	156.12(11)
N11B-Cu1-N1C	108.45(9)	N5B-Ag1-N11B	72.45(11)
N5A-C6A-C7A-S8A	2.9(3)	N5A-C6A-C7A-S8A	-179.9(3)
N5A-C6A-C7A-C11A	-176.0(2)	N5A-C6A-C7A-C11A	-0.5(6)
N5B-C6B-C7B-S8B	-170.24(19)	N5B-C6B-C7B-S8B	-179.3(3)
N5B-C6B-C7B-N11B	9.1(4)	N5B-C6B-C7B-N11B	-2.1(6)
X/Y	A: 85.3(2)<br>B: 75.4(2)	X/Y	A: 73.1(3)<br>B: 83.9(3)

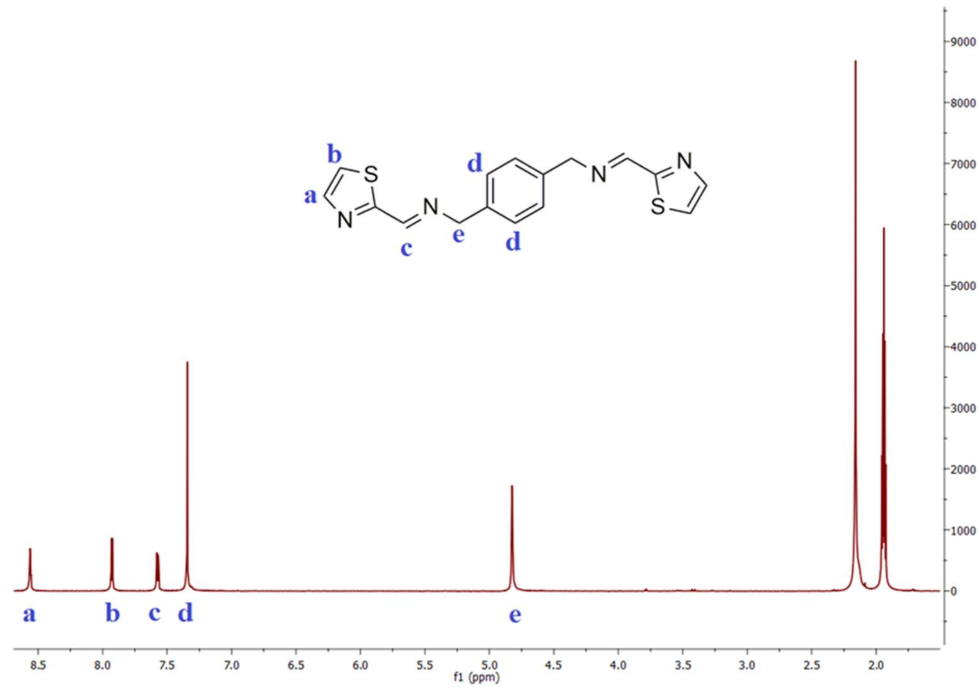

*Figure S 1 ^{1}H NMR spectrum of ligand **L1** in ACN-d3.*

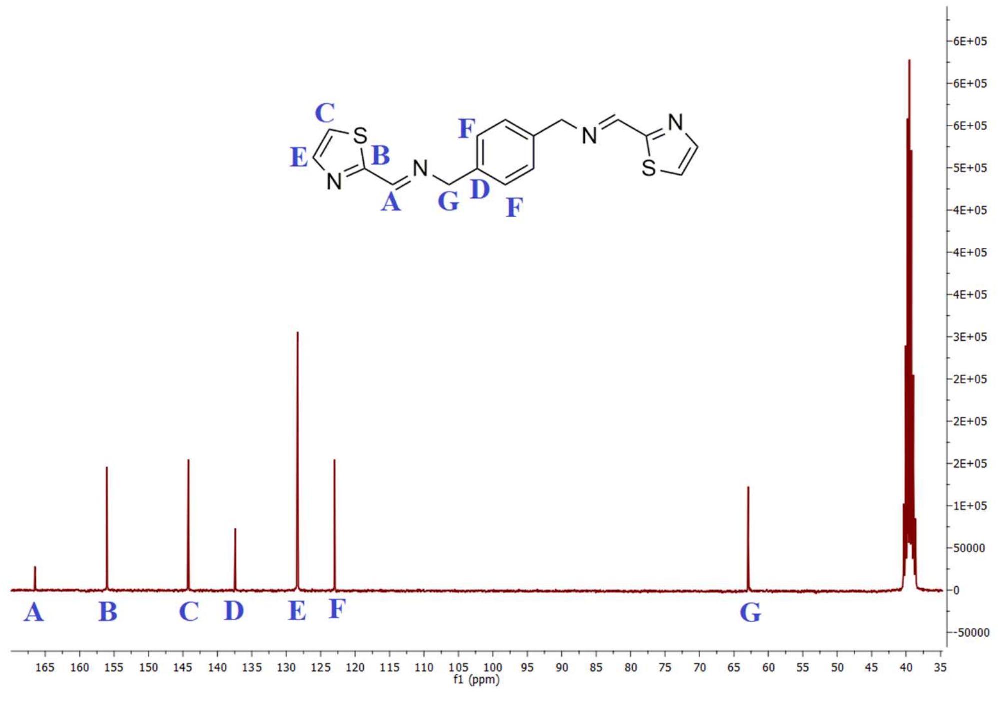

*Figure S 2 ^{13}C NMR spectrum of ligand **L1** in DMSO-d6.*

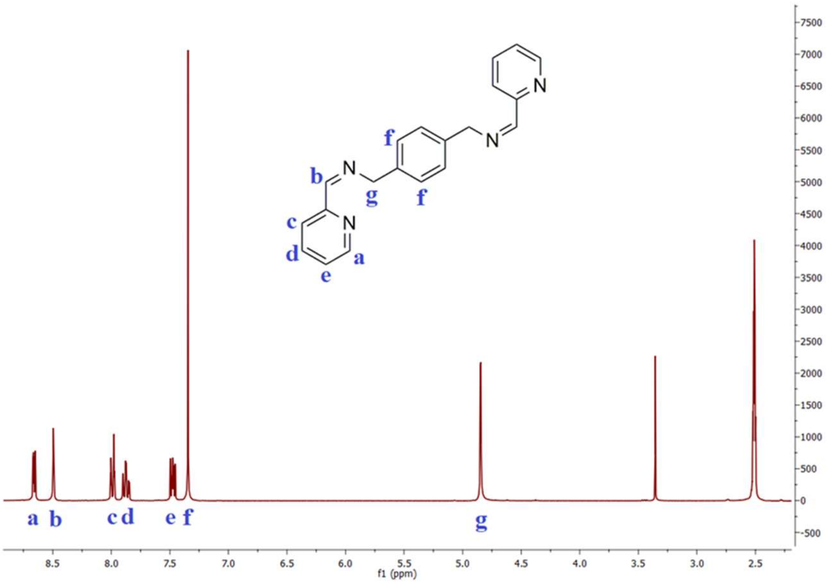

*Figure S 3 ^{1}H NMR spectrum of ligand **L2** in DMSO-d6.*

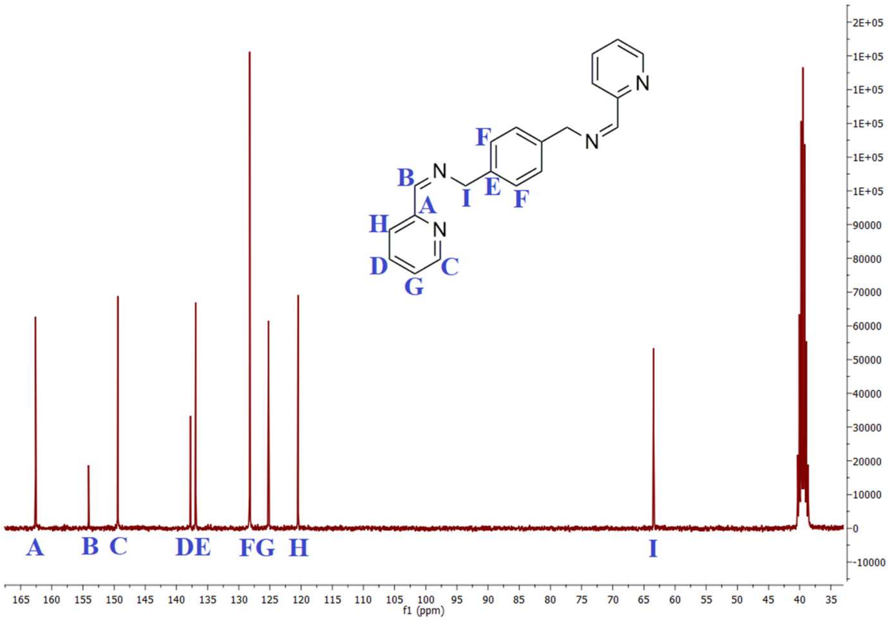

*Figure S 4 ^{13}C NMR spectrum of ligand **L2** in DMSO-d6.*

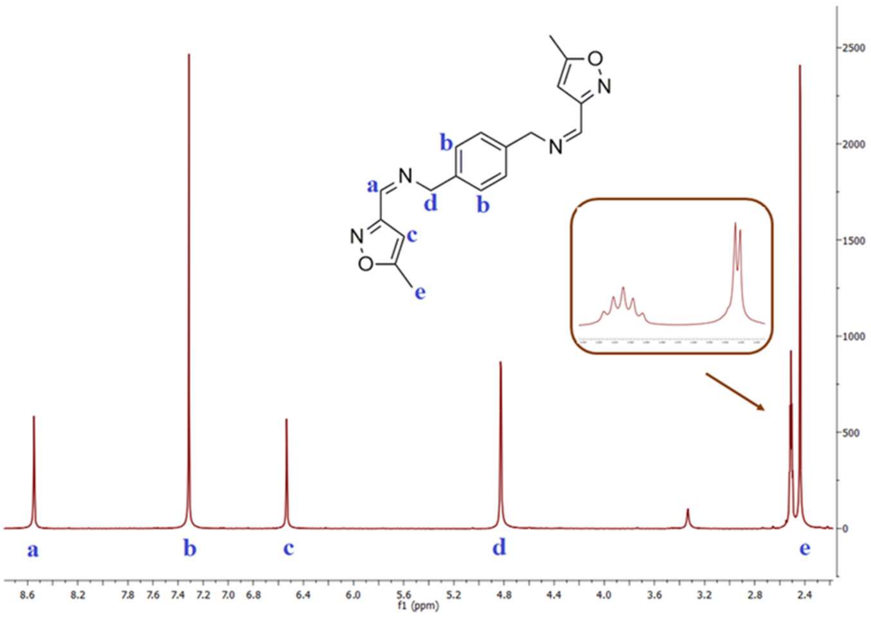

Figure S 5 ^{1}*H NMR spectrum of ligand* ***L3*** *in DMSO-d6.*

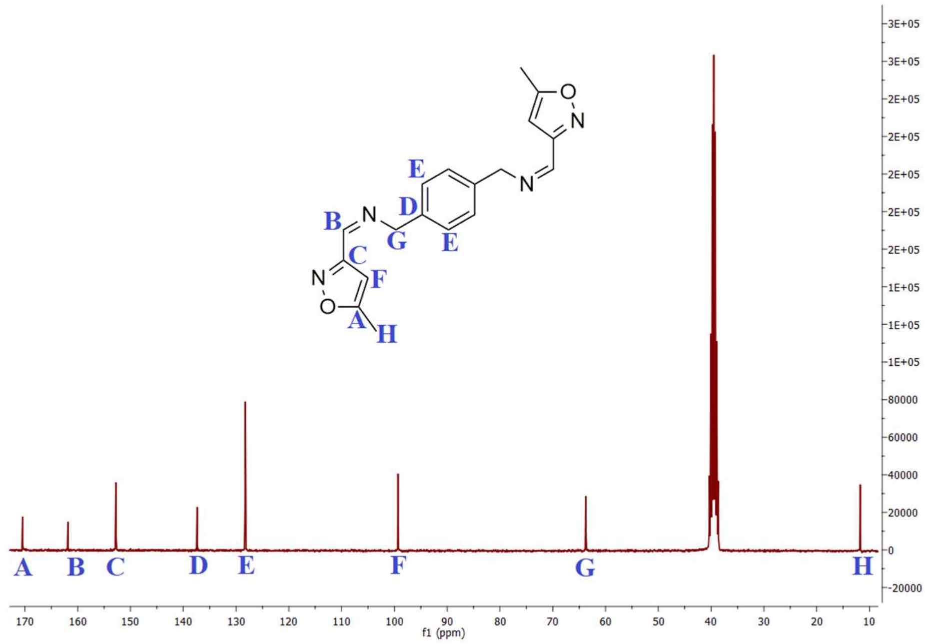

Figure S 6 ^{13}*C NMR spectrum of ligand* ***L3*** *in DMSO-d6.*

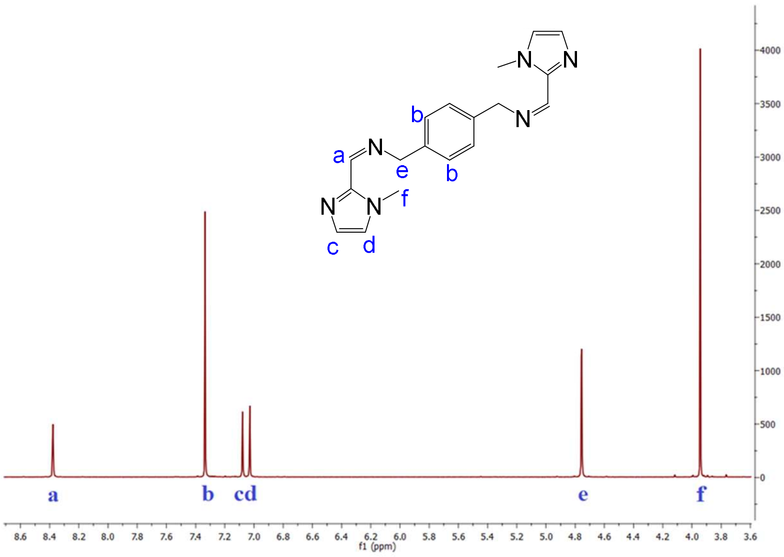

Figure S 7 ^{1}H NMR spectrum of ligand **L4** in ACN-d3.

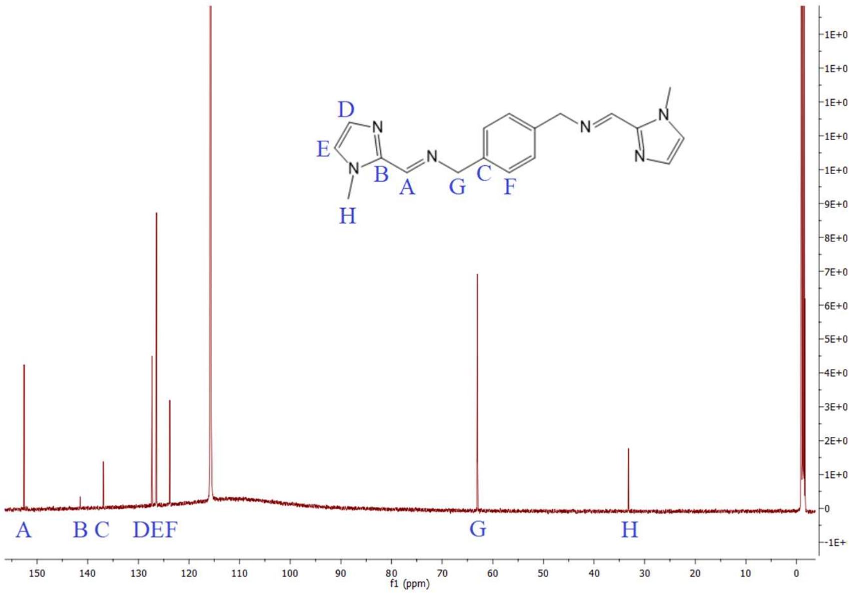

*Figure S 8 ^{13}C NMR spectrum of ligand **L4** in ACN-d3.*

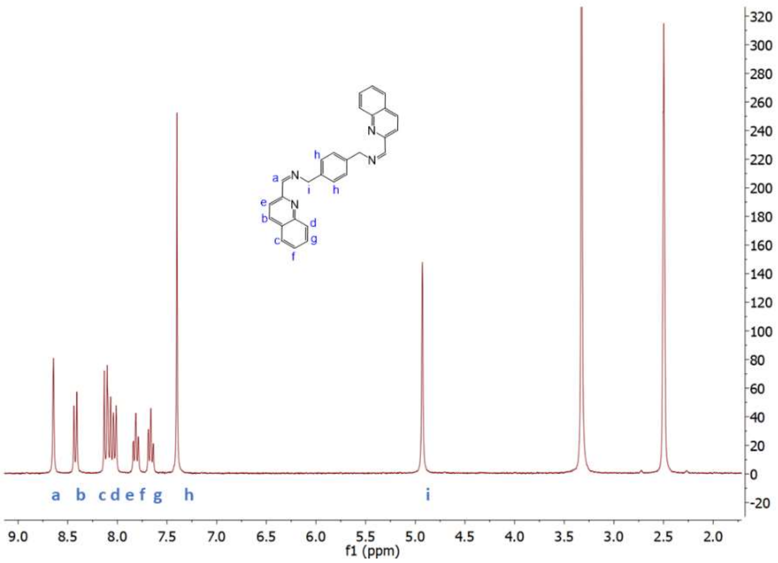

*Figure S 9 ¹H NMR spectrum of ligand **L5** in DMSO-d6.*

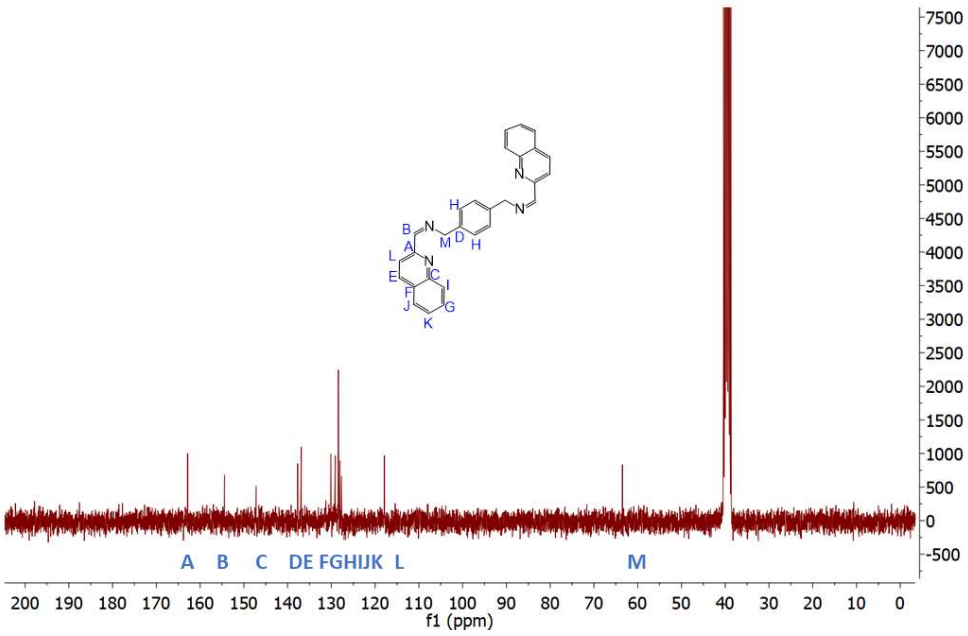

*Figure S 10 ¹³C NMR spectrum of ligand **L5** in DMSO-d6.*

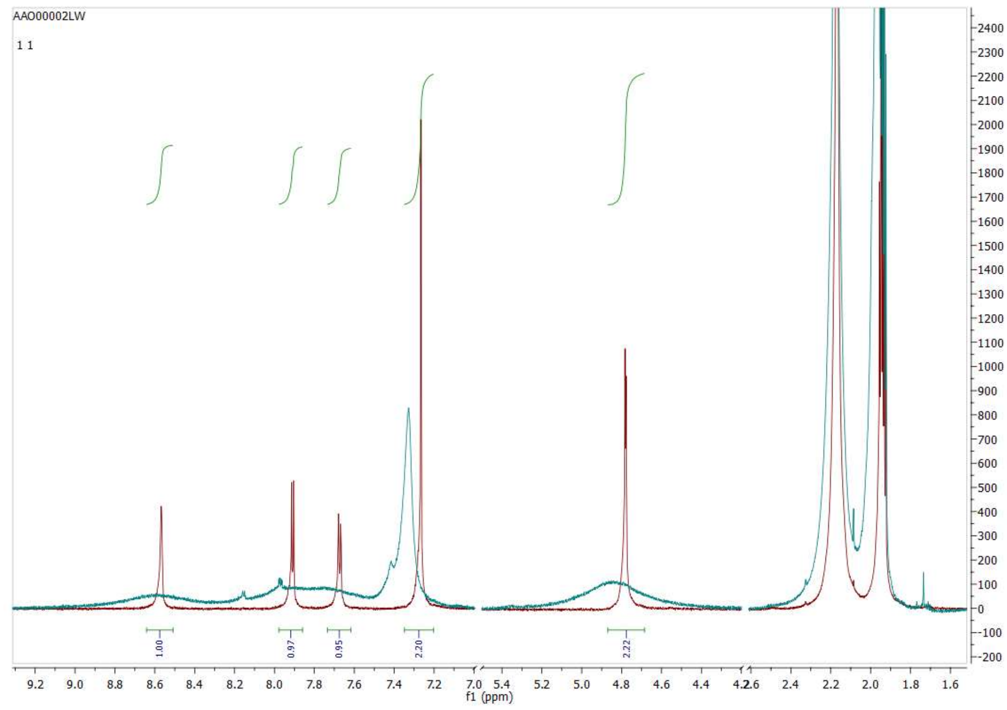

*Figure S 11 Superimposed ^{1}H NMR spectra of **L1** and coordination polymer {[Cu**L1**]PF_6}$_n$ in ACN-d3.*

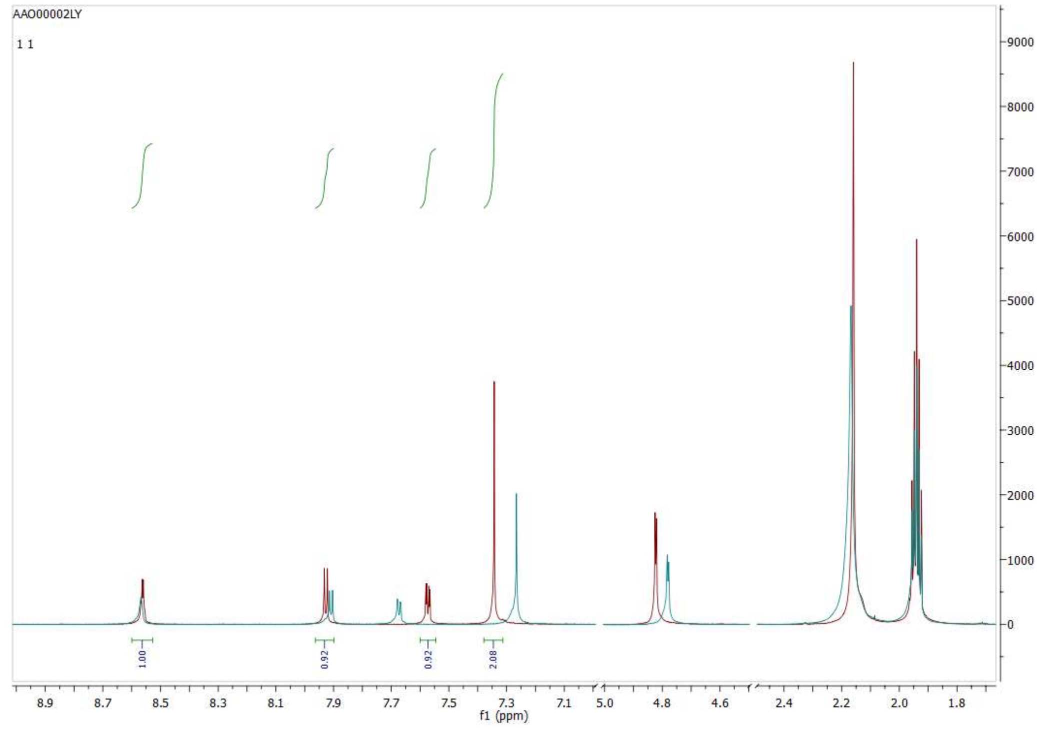

*Figure S 12 Superimposed ^{1}H NMR spectra of **L1** and coordination polymer {[Ag**L1**]BF_4}$_n$ in ACN-d3.*

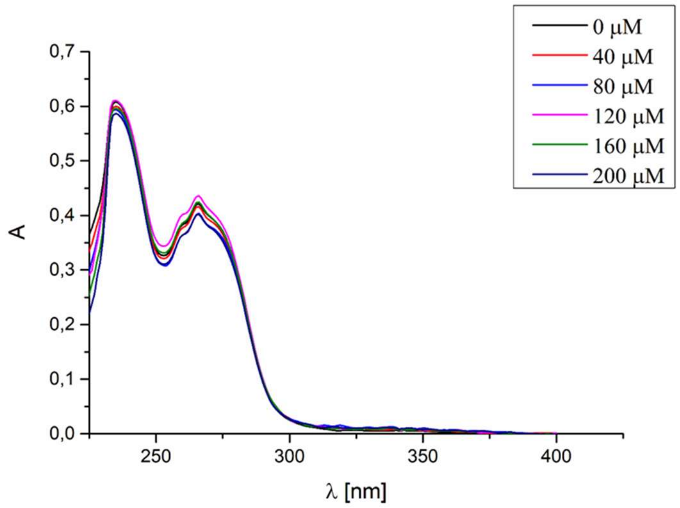

*Figure S 13 Spectrophotometric titration of **L2** ligand with CT-DNA.*

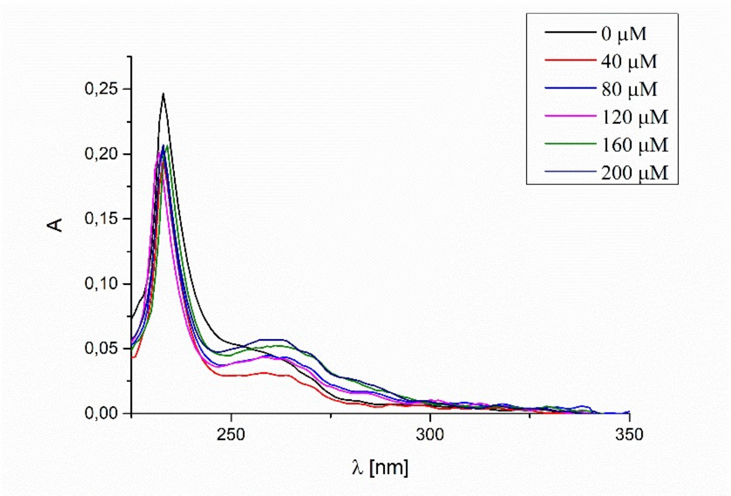

*Figure S 14 Spectrophotometric titration of **L3** ligand with CT-DNA.*

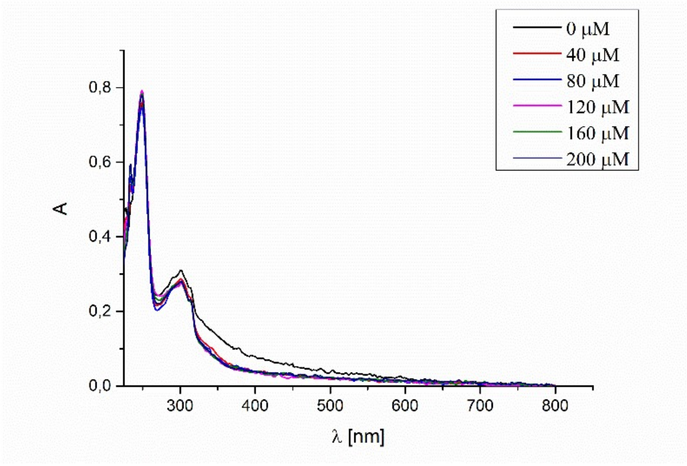

*Figure S 15 Spectrophotometric titration of **L4** ligand with CT-DNA.*

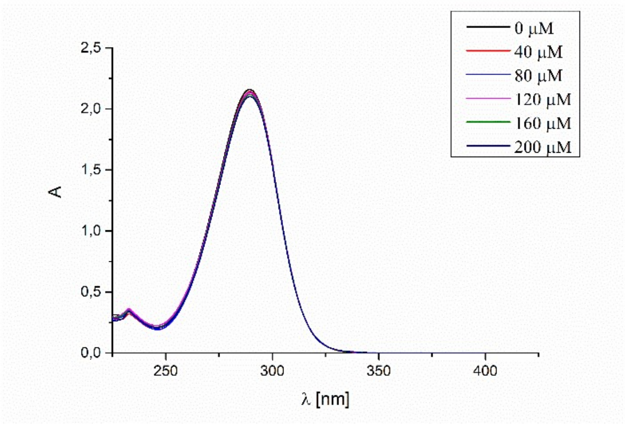

*Figure S 16 Spectrophotometric titration of **L5** ligand with CT-DNA.*

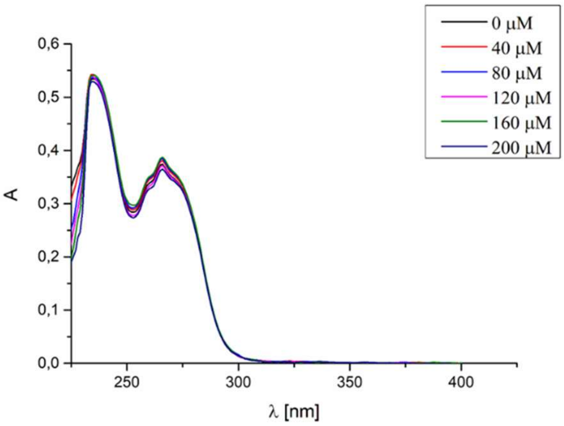

*Figure S 17 Spectrophotometric titration of **L2** ligand with RNA.*

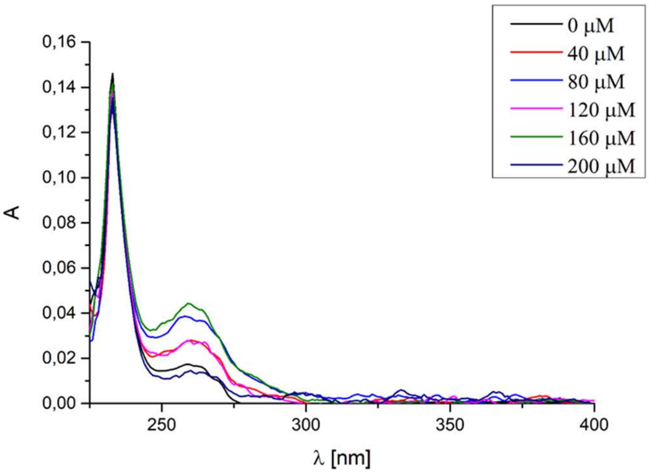

*Figure S 18 Spectrophotometric titration of **L3** ligand with RNA.*

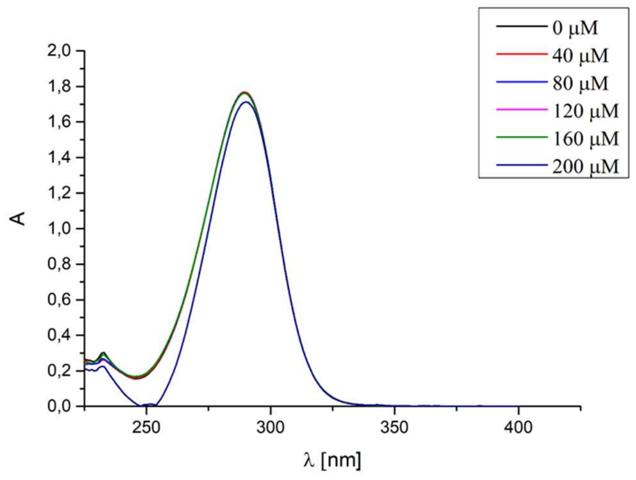

*Figure S 19 Spectrophotometric titration of **L4** ligand with RNA.*

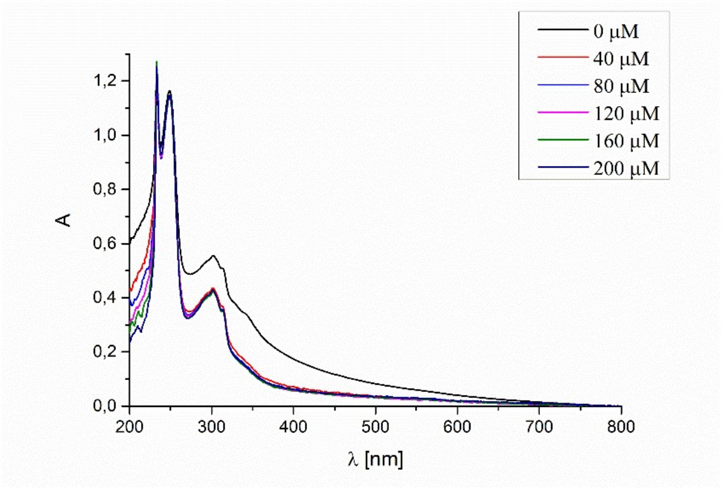

*Figure S 20 Spectrophotometric titration of **L5** ligand with RNA.*
